# Flagellum-deficient *Pseudomonas aeruginosa* is more virulent than non-motile but flagellated mutants in a cystic fibrosis mouse model

**DOI:** 10.1128/spectrum.01325-24

**Published:** 2024-09-09

**Authors:** Dina A. Moustafa, Kayla M. Fantone, Samantha L. Tucker, Nael A. McCarty, Arlene A. Stecenko, Joanna B. Goldberg, Balázs Rada

**Affiliations:** 1 Division of Pulmonology, Asthma, Cystic Fibrosis and Sleep, Department of Pediatrics, Emory University School of Medicine, Atlanta, Georgia, USA; 2 Department of Infectious Diseases, College of Veterinary Medicine, The University of Georgia, Athens, Georgia, USA; University of Texas Southwestern Medical Center, Dallas, Texas, USA

**Keywords:** cystic fibrosis, *Pseudomonas aeruginosa*, flagellum, motility, infection, inflammation, neutrophil, neutrophil elastase

## Abstract

Loss of the flagellum marks the pathoadaptation of *Pseudomonas aeruginosa* to the cystic fibrosis (CF) airway environment during lung disease. Losing the flagellum is advantageous to the bacterium as the flagellum can be recognized by immune cells. The primary purpose of the flagellum is, however, to provide motility to the bacterium. Our goal was to determine whether the loss of flagellar motility or the loss of flagellum expression contributes to *P. aeruginosa* lung infection in CF. To address this, wild-type and gut-corrected FABP-human cystic fibrosis transmembrane conductance regulator (hCFTR) mice deficient in the murine *Cftr* gene were infected intratracheally with lethal doses of wild-type or flagellum-deficient *P. aeruginosa*. While there was no significant difference in the survival of wild-type mice after infection with either of the bacterial strains, a significantly higher mortality was observed in FABP-hCFTR mice infected with flagellum-deficient *P. aeruginosa*, compared to mice infected with their flagellated counterparts. When FABP-hCFTR mice were infected with isogenic, motility-deficient flagellated mutants, animal survival and lung bacterial titers were similar to those observed in mice infected with the wild-type bacterium. Airway levels of neutrophils and the amount neutrophil elastase were similar in mice infected with either the wild-type bacteria or the flagellum-deficient *P. aeruginosa*. Our results show that FABP-hCFTR mice have a different response to flagellum loss in *P. aeruginosa* compared to wild-type animals. The loss of flagellum expression, rather than the loss of motility, is the main driver behind the increased virulence of flagellum-deficient *P. aeruginosa* in CF. These observations provide new insight into *P. aeruginosa* virulence in CF.

**IMPORTANCE:**

*Pseudomonas aeruginosa*, a major respiratory pathogen in cystic fibrosis, is known to lose its flagellum during the course of infection in the airways. Here, we show that the loss of flagellum leads to a more enhanced virulence in Cftr-deficient cystic fibrosis mice than in control animals. Loss of flagellum expression, rather than the loss of flagellar swimming motility, represents the main driver behind this increased virulence suggesting that this appendage plays a specific role in *P. aeruginosa* virulence in cystic fibrosis airways.

## INTRODUCTION

*Pseudomonas aeruginosa* is an opportunistic pathogen that typically does not infect healthy individuals and mainly causes infections in immunocompromised patients (1). *P. aeruginosa* has been a dominant respiratory pathogen in people with cystic fibrosis (PwCF) (2) contributing to increased morbidity and mortality (2). Despite the positive impact of the recently available highly effector modulator therapy on CFTR function, bacterial respiratory infections with *P. aeruginosa* remain a major cause of morbidity and mortality for PwCF (3). Critically, the factors leading to the unique susceptibility of the cystic fibrosis (CF) lung (and not other organs in CF arguing against a generalized immune dysfunction) are not totally understood.

The flagellum is a characteristic and important cell organelle of *P. aeruginosa* that provides the ability of the bacterium to swim. This flagellum-driven motility is important for the establishment of new bacterial communities where nutrients are sufficient. A flagellum is typically expressed in environmental isolates of *P. aeruginosa* and early clinical isolates found in PwCF (4, 5). Along with the induction of mucoidy and defective lipopolysaccharide, the loss of the flagellum is one of the characteristic changes accompanying the pathoadaptation of *P. aeruginosa* in CF airways (5–8). In PwCF with chronic *P. aeruginosa* infection, the bacterium mainly exists in biofilms, which selects for flagellar mutants (9).

The receptor for detecting the extracellular bacterial flagellin is Toll-like receptor 5 (TLR5) (10, 11). The flagellin-TLR5 interaction is a major player in airway inflammation in CF (12). TLR5 is typically expressed on innate immune cells (neutrophils, dendritic cells) and airway epithelial cells (10, 11, 13). It has been assumed that the bacterial flagellum influences the host’s immune system exclusively by binding to its receptor, TLR5. Emerging *in vitro* data, however, indicate that flagellum-fueled swimming motility also plays an important role in interactions of *P. aeruginosa* with the host, in a TLR5-independent fashion. We have shown that swimming motility, not flagellum expression, is important for the stimulation of human neutrophils *in vitro* to release neutrophil extracellular traps (NETs) (14). Flagellar motility, not flagellum expression, also has been shown by others to stimulate the inflammasome and to mediate phagocytosis (15–17). These results propose a more complex mechanism, by which the flagellum participates in *P. aeruginosa* interaction with the host: (i) disadvantageous interactions for the bacterium mediated by the direct binding of flagellum to TLR5 and swimming motility stimulating NETs, and/or (ii) advantageous interactions where flagellum-mediated motility drives movement toward nutrient-rich environments and establishment of new communities.

The contributions of flagellum and motility to *P. aeruginosa* pathogenesis have not been studied in a CF animal model *in vivo*. Therefore, here, we investigated how the loss of the flagellum affects *P. aeruginosa* virulence in CF by studying flagellum-deficient and flagellar motility-deficient bacteria in CF mice.

## MATERIALS AND METHODS

### Mice

Cftr^tm1Unc^ Tg(FABP-hCFTR)1Jaw/J (here referred to as FABP-hCFTR mice) mice were bred and maintained at Emory University under the supervision of Division of Animal Resources protocol PROTO201800103. These mice are bitransgenic harboring the FABP-hCFTR transgene, with inserted rat fatty acid-binding protein 2 (FABP2) promoter directing expression of human CFTR (ATP-binding cassette sub-family C, member 7) gene allowing for high expression of the transgene throughout the small intestine (18). Male heterozygotes were bred with female heterozygotes. Genotyping of mice was performed by TransnetYX with genotyping carried out to confirm the deletion of murine *Cftr* and the presence of the human transgene.

### *P. aeruginosa* strains

All the *P. aeruginosa* strains used in this study were derived from the parental *P. aeruginosa* PAK that was obtained from Pathogenesis Corporation (Seattle, WA, USA) and referred to as wild-type (WT) in the manuscript. The isogenic (PAK), flagellum-deficient mutant *flgC1*::Tn*5* (referred to as “flgC”) was also obtained from the same source (Pathogenesis Corporation, Seattle, WA, USA). The flagellar motor mutant strains were also generated on the PAK background as described before (19). Briefly, in-frame deletion mutants of *motAB* and *motD* (in mot16) or *motB* and *motCD* (in mot50) were constructed, and flagellum expression and flagellar swimming motility were confirmed (14, 20). *P. aeruginosa* strains used in this work are listed in Table 1 (21, 22). *P. aeruginosa* strains were cultured in Lysogeny Broth (LB), washed twice in PBS, and resuspended in calcium- and magnesium-containing Hank’s Balanced Salt Solution (HBSS). Bacterial cultures were set to an optical density (OD) = 0.6 at 600 nm in 96-well microplates measured using a Varioskan Flash microplate reader (ThermoScientific, Waltham, MA, USA). This OD value corresponds to a bacterial density of 10^9^ /mL, as determined by serial dilutions and colony forming unit (CFU) assays (23, 24).

**TABLE 1:** Bacterial strains used in this study

Bacterial strain	Description	Flagellum expression	Swimming motility	References
PAK	Wild type	+	+	Pathogenesis Corp.
PAK Mot16	motB::Tn5 (Tetr) ?motCD::Genr	+	-	(21)
PAK Mot50	motAB::Tetr motD::Genr	+	-	(21)
PAK flgC	flgC1::Tn5(Tetr)	-	-	Pathogenesis Corp. (AKA 056E02)

### Murine model of pneumonia

The animal studies have been approved by the Institutional Animal Care and Use Committee (IACUC) of Emory University (approved protocol number DAR-2003045-ENTRPR-N). The studies were carried out in strict accordance with established guidelines and policies at Emory University School of Medicine, and recommendations in the Guide for Care and Use of Laboratory Animals, as well as local, state, and federal laws.

*P. aeruginosa* strains were grown in LB at 37°C for 16 h. Subsequently, the cultures were diluted 1:100 to an OD at 600 nm (OD_600_) of 0.05 and re-grown in LB for 4–6 h until OD_600_ = 0.5. Bacteria were washed, resuspended in PBS, and adjusted spectrophotometrically to obtain the indicated infection doses in a volume of 50 µL. Eight- to 12-week-old ^-^ FABP-hCFTR mice and WT litter mates (age- and sex-matched) were anesthetized by intraperitoneal injection of 0.2 mL of a mixture of ketamine (10 mg/mL) and xylazine (0.5 mg/mL). Mice were infected by non-surgical intratracheal instillation (25, 26) of dilutions of indicated *P. aeruginosa* strains. To perform non-surgical intratracheal infection, the tongue was pulled out with a blunt ended forceps in anesthetized mice and held on the side before a 1 mL syringe fitted with a sterile, bent gavage needle containing the inoculum was introduced into the trachea until the bend in the needle is by the front of incisors (25, 26). The plunger was pushed evenly to deliver the inoculum into the trachea without bubbles (25, 26). The needle was immediately pulled out to avoid suffocation, and the animals were held in an upright position to allow the inoculum to be inhaled into the lungs (25). For survival experiments, mice were monitored daily for a period of 96 h. For fixed end-point experiments, mice were euthanized at 18 h post-infection. Lungs were collected aseptically, weighed, and homogenized in 1 mL of PBS. Tissue homogenates were serially diluted and plated on *Pseudomonas* isolation agar for CFU determination. Bronchoalveolar lavage fluids (BALs) were collected from a parallel group of mice.

### Flow cytometry

To determine whether neutrophils are present in the lungs of mice, cells recovered from collected BAL fluids were counted and stained. Cells were first resuspended in 1× PBS and stained with Zombie Aqua fixable dye (1:1,000, BioLegend, San Diego, CA, USA) on ice for 15 min. The cells were then washed and resuspended in 1% bovine serum albumine (BSA) in 1× PBS. All the steps were performed on ice and protected from light. The cells were then blocked with TruStain FcX (Biolegend) for 10 min. The antibodies (CD11b (PECy7 cat#101215), CD115 (APC cat#135509), and Ly6G (AF488 cat#127625), Biolegend) were then added to the cells and incubated for 1 h. Following incubation, cells were washed, fixed with stabilizing fixative (BD Biosciences, San Jose, CA, USA), and analyzed with the NovoCyte Flow Cytometer (Agilent Technologies, Santa Clara, CA, USA) with the NovoExpress software at the University of Georgia, College of Veterinary Medicine Cytometry Core. Live neutrophils were characterized as zombie^-^, CD11b^+^, CD115^-^, and Ly6G^+^.

### ELISA

Concentration of murine neutrophil elastase (NE) was quantitated by a commercial ELISA kit according to the manufacturer’s suggestions (R&D Systems, Minneapolis, MN, USA) as previously described (27). Results were expressed as pg/mL in BAL fluids.

### Statistical analysis

The results of survival studies were presented using Kaplan–Meier survival curves and were analyzed by the log-rank test. The numbers of viable bacteria obtained in lung homogenates were analyzed using one-way analysis of variance (ANOVA) and were compared using the Kruskal–Wallis test for comparison. Results of neutrophils and NE in murine airway samples were compared by one-way ANOVA and Tukey’s multiple comparison test. Differences were considered significant if *P < 0.05; **P < 0.01; ***P < 0.001. Data were analyzed with GraphPad Prism software version 10.0.2.

## RESULTS

### Flagellum-deficient *P. aeruginosa* is more virulent in a CF mouse model than its flagellated counterparts

**FIG 1:**
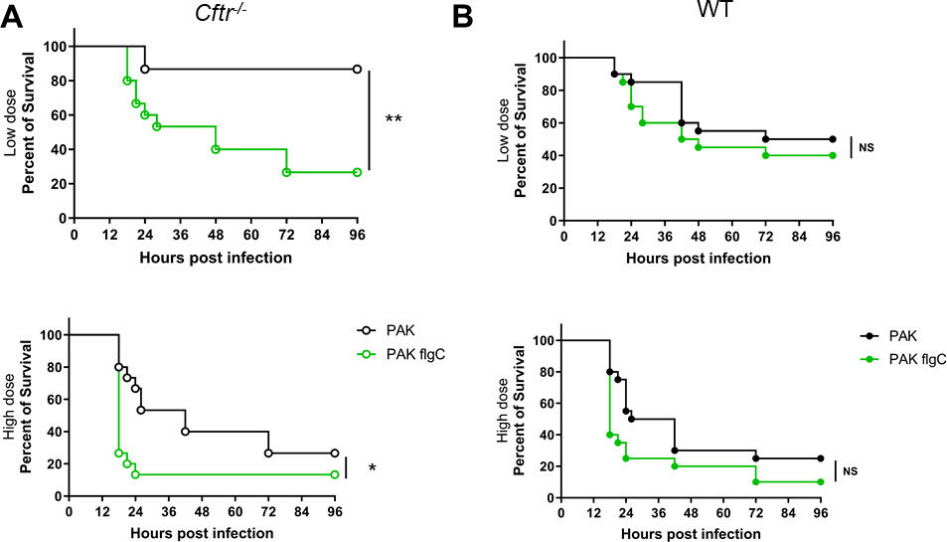
The lack of a flagellum increases *P. aeruginosa* virulence in FABP-hCFTR mice. (A) FABP-hCFTR (*n* = 15) and (B) WT littermate mice (*n* = 20) were infected intratracheally with a low dose (1.2–1.5 × 10^6^ CFU/mouse) or high dose (~2.5×10^6^ CFU/mouse) of *P. aeruginosa* PAK or PAK flgC strains and were monitored for survival for 4 days. Results are presented by Kaplan–Meier survival curves and were analyzed by log-rank test. *P < 0.05; **P < 0.01. ns, nonsignificant.

The loss of flagellum represents a typical change that accompanies *P. aeruginosa* pathoadaptation to the airways in PwCF (5–8). We, therefore, first compared lung infection caused by a flagellum-deficient *P. aeruginosa* strain (flgC) to that caused by the WT, flagellated bacterium (PAK) in FABP-hCFTR mice (18, 28). The lack of flagellum expression and swimming motility in the flgC strain and their presence in the parental strain (PAK) have been previously characterized (14, 20). Mice were infected intratracheally with one of the two different doses, low (1.2–1.5 × 10^6^ CFU/mouse) or high (2.5 × 10^6^ CFU/mouse), of *P. aeruginosa*, and survival was followed for up to 4 days post-infection. Lung infection in FABP-hCFTR mice with the flagellum-deficient *P. aeruginosa* resulted in significantly higher mortality than that was caused by the WT flagellated strain (Fig. 1A). This effect was observed in both the high and the low infectious doses in FABP-hCFTR animals (Fig. 1A). Interestingly, the significant difference in survival observed early in FABP-hCFTR mice between flagellated and flagellum-deficient bacteria disappeared through time and became nonsignificant when WT mice were tested in an identical fashion (Fig. 1B). These data indicate that WT mice might not represent the most appropriate model for our studies, and the lack of flagellum expression significantly increases *P. aeruginosa* virulence in CF mice, an observation documented in PwCF. Thus, FABP-hCFTR mice were used in experiments mentioned throughout in the rest of the manuscript.

### Loss of flagellum expression, not that of swimming motility, drives the increased virulence of flagellum-deficient *P. aeruginosa* in FABP-hCFTR mice

After having established that the loss of bacterial flagellum enhances *P. aeruginosa* virulence in FABP-hCFTR mice (Fig. 1A), next we aimed at determining whether the loss of flagellum expression or flagellar motility is more important in the increased mortality observed in infections by flagellum-deficient bacteria. To address this question, two isogenic *P. aeruginosa* PAK mutants were used that are nonmotile but express flagellum on their surface. These mutants, mot16 and mot50, are deficient in genes responsible for the rotation of the flagellum, not for flagellum assembly and expression (20) (Table 1). The *P. aeruginosa* flagellum is powered by a complex bacterial motor that consists of several proteins encoded by two sets of homologous motor genes: (i) *motA*, *motB* and (ii) *motC*, *motD* (20, 29). Disruption of both loci is needed to abolish swimming motility in the bacterium (14, 20, 29). We previously characterized motility and flagellin production in these two PAK strains deficient in the following *mot* genes: strain LMP16 (Δ*motCD motB*, here referred to as mot16) and strain LMP50 (Δ*motAB motD*, here referred to as mot50) (14).

**FIG 2:**
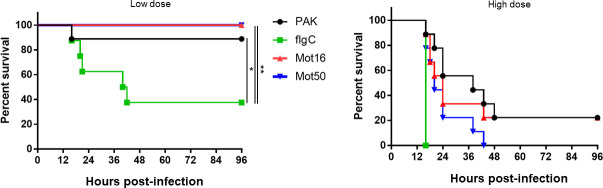
Loss of flagellum expression, not the loss of swimming motility, is the main contributor to increased virulence of flagellum-deficient *P. aeruginosa* in FABP-hCFTR mice. FABP-hCFTR mice (*n* = 9) were infected intratracheally with a low dose (1.2–1.5 × 10^6^ CFU/mouse) or a high dose (~2.5×10^6^ CFU/mouse) of *P. aeruginosa* PAK or indicated isogenic mutants and were monitored for survival for 4 days. Results are presented by Kaplan–Meier survival curves and were analyzed by log-rank test. *P < 0.05; **P < 0.01.

FABP-hCFTR mice were infected with WT (flagellum-expressing, motile, PAK), flagellar motility-deficient (mot16 and mot50) and flagellum-deficient, nonmotile (flgC) *P. aeruginosa* strains as described earlier (Fig. 1). Both low and high doses were tested. As expected, flagellum-deficient *P. aeruginosa* infection resulted in increased mortality compared to WT bacteria in FABP-hCFTR mice (Fig. 2). Infections with low doses of the nonmotile but flagellum-expressing mutants (mot16 and mot50) led to no mortality in FABP-hCFTR mice and thus were more similar to the WT bacterium. These data indicate that the loss of flagellum expression, not the loss of swimming motility, is the reason for the increased virulence of flagellum-deficient *P. aeruginosa* in CF (Fig. 2). This pattern became less clear when the high bacterial dose was used, and mortalities caused by mot16 and mot50 infections were not significantly different between WT or flagellum-deficient *P. aeruginosa* strains (Fig. 2). The pattern observed in survival kinetics is also reflected in the median survival times (Table 2). These results suggest that at earlier stages of *P. aeruginosa* infection (represented by the low infectious dose) flagellum expression, not swimming motility, is responsible for *P. aeruginosa* recognition and clearance by the immune system leading to decreased mortality caused by flagellum-deficient bacteria. Overall, our data suggest that the loss of flagellum expression, not the loss of swimming motility, is the major contributor to the increased virulence of flagellum-deficient *P. aeruginosa* in a lung infection murine model of CF.

**TABLE 2:** Median survival time of FABP-hCFTR mice infected with the parental PAK strain (WT) and its isogenic mutants

Strains	Low dose (h)	High dose (h)
WT	Undefined	38
flgC	41	16
Mot 15	Undefined	24
Mot 50	Undefined	20

### Loss of flagellum expression leads to an increased *P. aeruginosa* lung burden in FABP-hCFTR mice

**FIG 3:**
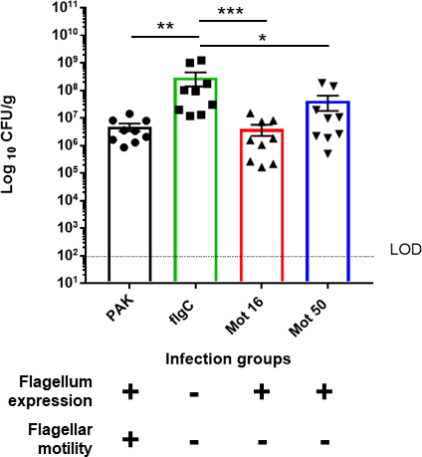
Loss of flagellum expression leads to increased *P. aeruginosa* lung burden in FABP-hCFTR mice. FABP-hCFTR mice (*n* = 9) were infected intratracheally with the low dose (1.2–1.5 × 10^6^ CFU/mouse) of *P. aeruginosa* PAK or indicated isogenic mutants. Bacterial loads in the lungs were assessed 18 h post-infection. All samples were plated for viable CFU on *Pseudomonas* isolation agar. Each point represents a single mouse. Data were analyzed by one-way ANOVA and were compared using Kruskal–Wallis test for significance. *P < 0.05; **P < 0.01; ***P < 0.001. Error bars represent the mean and SEM. The dashed line represents the limit of detection (LOD).

To determine whether the worsened survival of mice infected with flagellum-deficient *P. aeruginosa* associates with increased bacterial burden, *P. aeruginosa* colonization in the lungs of infected animals was determined. Bacterial lung loads in mice infected with flagellum-deficient *P. aeruginosa* flgC were significantly higher than those infected with the WT bacterium (Fig. 3), confirming that increased mortality associates with higher *P. aeruginosa* burden.

To assess whether the loss of flagellum expression or just the loss of motility was driving the higher bacterial lung burden in mice infected with flagellum-deficient *P. aeruginosa*, bacterial colonization was also determined in the lungs of mice infected with the flagellated, motility-deficient mutants, mot16 and mot50. Lung bacterial burden of mot16 and mot50 was significantly lower than that of flgC (Fig. 3). There were no significant differences in the bacterial burden between the WT bacterium, PAK, and the motility-deficient mutants (Fig. 3). These data suggest that the loss of flagellum expression, rather than the loss of motility, drives the high bacterial lung colonization observed in mice infected with flagellum-deficient *P. aeruginosa* (Fig. 3). Bacterial lung burden data correlate well with mortality results and both suggest a major role for the loss of flagellum expression in the increased virulence of flagellum-deficient *P. aeruginosa* in CF mice.

### Neutrophil recruitment is largely independent of bacterial flagellum

**FIG 4:**
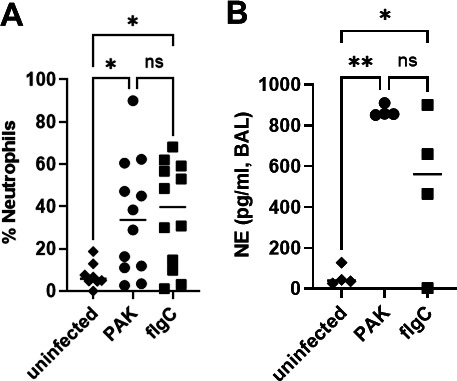
Bacterial flagellum does not affect airway levels of neutrophils or NE in *P. aeruginosa*-infected FABP-hCFTR mice. FABP-hCFTR mice (*n* = 4–12) were infected intratracheally with low dose (1.2–1.5 × 10^6^ CFU/mouse) of *P. aeruginosa* PAK or indicated isogenic mutants. The percentage of neutrophils and the amount of NE in the BAL were assessed 18 h post-infection in surviving animals by flow cytometry or ELISA, respectively. Each point represents a single animal. Data were analyzed by one-way ANOVA and Tukey’s multiple comparison test. *P < 0.05; **P < 0.01. Mean of each data set is shown..

Polymorphonuclear neutrophilic granulocytes (PMN, neutrophils) are essential to combat *P. aeruginosa*. Mammals lacking phagocytes or certain molecules of the innate immune system are susceptible to *P. aeruginosa* infection (30, 31). Neutrophils are present in large numbers in CF airways and represent major drivers of chronic inflammation but are unable to clear a select group of pathogens including *P. aeruginosa* (32). Therefore, we measured the percentage of neutrophils in the bronchoalveolar lavage of mice infected with WT or flagellum-deficient *P. aeruginosa* flgC to determine whether the loss of flagellum affects neutrophil airway recruitment in CF mice. Infections by either of the two bacterial strains led to significantly increased numbers of neutrophils in the lungs of FABP-hCFTR mice but no flagellum-dependent difference was observed (Fig. 4A). Thus, the flagellum does not seem to be a major player in recruiting neutrophils to the airways in CF mice following *P. aeruginosa* infection.

### Neutrophil elastase airway levels remain unaffected by *P. aeruginosa* flagellum

As the NE levels in the sputa correlate with lung disease severity in PwCF (33–37), we were curious to know whether NE levels are affected by bacterial flagellum. NE levels were significantly higher in the airways of mice infected with WT or flagellum-deficient *P. aeruginosa* flgC compared to uninfected animals (Fig. 4B). However, there was no significant difference in airway NE levels between mice infected with either the WT or flgC bacterium (Fig. 4B). Thus, the presence of a flagellum on *P. aeruginosa* does not appear to affect neutrophil recruitment or NE release in the airways of CF mice.

## DISCUSSION

*P. aeruginosa* strains that colonize the airways of PwCF are typically motile but usually convert to a nonmotile phenotype as lung disease advances (38). The loss of flagellum has been associated with the establishment of *P. aeruginosa* infection in CF and other diseases (6, 39). Interestingly though, flagellin was detected in expectorated sputa of PwCF regardless of whether they were colonized with mucoid or non-mucoid *P. aeruginosa* strains. This suggest that flagellum-expressing bacteria can always be present in the airways of PwCF with *P. aeruginosa* infection and could have a relevant role in lung disease (40). Immune cells are equipped with receptors specific for the recognition of bacterial flagellum in the extracellular/phagosomal space or in the cytosol (41). The surface receptor for the detection of extracellular bacterial flagellin is TLR5, while flagellin in the cytosol is sensed by nucleotide oligomerization domain (NOD)-like receptor CARD domain-containing 4 (NLRC4) (42). In addition, motile *P. aeruginosa* has much higher chances to encounter host immune cells through their continuous motion than nonmotile bacteria. Additionally, a motile bacterium could seek out nutrient-rich environments and establish more bacterial colonies. Thus, there may be conflicting survival interests of *P. aeruginosa* to downregulate flagellum expression (41).

The role of the flagellum in murine models of *P. aeruginosa* lung infection has been studied previously. In WT, C57BL/6 mice infected with a flagellin-deficient *P. aeruginosa* strain (PAK-C), survival was better than in mice infected with the flagellum-expressing, WT bacterial strain (PAK) (43). Flagellin (*fliC*)-deficient *P. aeruginosa* PAK was less virulent in neonatal BALBc/ByJ mice than the flagellated parental strain (44). These previous reports using non-CF mice indicate a role of flagellin in *P. aeruginosa* virulence in lung infection. In contrast to this, our observations made in WT mice indicate no significant role of flagellin in *P. aeruginosa*-induced mortality. These contradictory reports could result from using different mouse strains, age at infection, or routes of administration.

To our knowledge, no prior study investigated the role of the flagellum in *P. aeruginosa* lung infection in a CF mouse model. Our findings are in contrast to those reported in non-CF mice (43, 44) but are consistent with the observation that the loss of flagellum is important in establishing *P. aeruginosa* lung infection in PwCF (39).

TLR5 signaling has been reported to be hyper-responsive in porcine CF airways (45). TLR5 also acts as a modifier gene in CF (12). A CF airway epithelium that overreacts to the presence of flagellin via TLR5 could explain the importance of TLR5 signaling and biology in CF and support our findings. Airway administration of flagellin has been shown to decrease the severity of *P. aeruginosa*-stimulated pneumonia in mice (46). Airway epithelial uptake of flagellin initiated proinflammatory signaling (47). These and other Cftr-dependent mechanisms present in WT mice could enable an airway environment that promotes clearance of *P. aeruginosa* mainly by flagellum-independent mechanisms. In the absence of *Cftr*, however, these mechanisms would fail or be impaired and flagellum recognition and targeting would become more relevant in *P. aeruginosa* clearance from the airways of FABP-hCFTR mice (48).

Our studies addressing the role that the flagellum plays in pathogenesis have been enabled by bacterial mutants that express the flagellum but are unable to rotate it (14, 17, 20). We also have utilized such mutants in our current studies to learn about the role of flagellum expression versus flagellar motility in CF mice. These flagellated but nonmotile *P. aeruginosa* strains represent elegant, genetic experimental tools that are superior over approaches that kill flagellated bacteria by various methods (14, 20).

Neutrophils have an essential role in the immune response to *P. aeruginosa*. The lack of neutrophils makes a host susceptible to *P. aeruginosa* infection (30, 31, 49). Neutropenia, caused by chemotherapy, HIV infection, or autoimmune disorders, predisposes patients to *P. aeruginosa* pneumonia (19, 50, 51). The airways of PwCF contain large numbers of neutrophils. CF sputum neutrophil counts, concentrations of myeloperoxidase, NE, and neutrophil chemoattractants correlate with lung disease progression (52–55). Earlier studies reported the need for TLR5 for a maximal immune response against the flagellum (56–59). More recent studies by our group dissected the role of flagellar motility vs flagellum expression (and binding to its receptors including TLR5) in *P. aeruginosa* infections. We found that *P. aeruginosa* interacts with neutrophils *in vitro* in a flagellum-dependent but TLR5- and NLRC4-independent fashion (14). Only flagellated and motile, WT bacteria were phagocytosed by neutrophils and induced the production of reactive oxygen species and NETs in neutrophils (14). Flagellated but nonmotile or flagellum-deficient *P. aeruginosa* strains were all significantly impaired in eliciting these neutrophil effector responses *in vitro* (14). Studies done by other groups further pointed to the relevance of flagellar motility in *P. aeruginosa* virulence. Evasion of phagocytosis of *P. aeruginosa* is mediated by the loss of swimming motility and does not depend on flagellum expression (16). Flagellar motility, not flagellum expression, was reported to initiate signaling pathways that mediate *P. aeruginosa* engulfment in phagocytes (15). Flagellar motility is also important for inflammasome activation (17). These *in vitro* studies suggest a complex role of flagellum in the interactions between *P. aeruginosa* and innate immune cells.

Our work identified an overall primary role for the loss of flagellum expression over the loss of flagellar motility in driving increased virulence of flagellum-deficient *P. aeruginosa* in CF mice *in vivo*. The role of flagellum expression in *P. aeruginosa* virulence was further confirmed by the lung bacterial colonization data. These findings add to the increasing body of literature studying the complex *in vivo* roles the flagellum plays in CF lung disease pathogenesis.

Our data indicate that *P. aeruginosa* lung infection leads to neutrophil recruitment in the airways. This is consistent with other reports done with *P. aeruginosa*-infected mice (60, 61). Flagellum expression does not significantly affect airway levels of neutrophils suggesting that while the flagellum is associated with worse clinical outcomes in *P. aeruginosa*-infected CF mice, its mechanism is unlikely to involve neutrophil airway recruitment. Airway NE levels also were elevated in infected mice but were not different between mice infected with flagellated and motile or flagellum-deficient *P. aeruginosa*, although there was more variation in the response of the flagella-deficient strain.

Our study has limitations. The *in vivo* mouse model used in our study leads to rapid mortality in FABP-hCFTR mice that does not accurately represent the slower, chronic *P. aeruginosa* infection dynamics in the airways of PwCF. The model is most appropriate to study the onset of *P. aeruginosa* infections in CF airways and to better understand the relevance of *Cftr*-dependent and -independent mechanisms of *P. aeruginosa* clearance from the lung. The aim of this study was to deliver proof-of-concept data regarding the effect of loss of flagellar motility or expression on *P. aeruginosa* infection in a CF mouse model. Overall, our study reports an essential role of the loss of the flagellum in determining the outcomes of lethal *P. aeruginosa* lung infection in CF mice. Our work determined that the loss of flagellum expression was more important than the loss of swimming motility in the increased lung disease pathogenesis in FABP-hCFTR mice infected with flagellum-deficient *P. aeruginosa*. This knowledge leads to a better understanding of *P. aeruginosa* infection and lung disease pathogenesis in CF.

## Data Availability

The data of this manuscript will be available upon publication from the senior author of the study upon request.
